# Development and validation of a nomogram with an epigenetic signature for predicting survival in patients with lung adenocarcinoma

**DOI:** 10.18632/aging.104090

**Published:** 2020-11-18

**Authors:** Jiao Wang, Li He, Yunliang Tang, Dan Li, Yuting Yang, Zhenguo Zeng

**Affiliations:** 1 Department of Endocrinology and Metabolism, The First Affiliated Hospital of Nanchang University, Nanchang 330006, Jiangxi, China; 2 Department of Pathology, Jingdezhen First People's Hospital, Jingdezhen 333000, Jiangxi, China; 3 Department of Rehabilitation Medicine, The First Affiliated Hospital of Nanchang University, Nanchang 330006, Jiangxi, China; 4 Department of Respiratory and Critical Care Medicine, The First Affiliated Hospital of Nanchang University, Nanchang 330006, Jiangxi, China; 5 Department of Critical Care Medicine, The First Affiliated Hospital of Nanchang University, Nanchang 330006, Jiangxi, China

**Keywords:** lung adenocarcinoma, epigenetic signature, prognosis, nomogram

## Abstract

Epigenetic factors play crucial roles in carcinogenesis by modifying chromatin architecture. Here, we established an epigenetic biosignature-based model for examining survival in patients with lung adenocarcinoma (LUAD). We retrieved gene-expression profiles and clinical data from The Cancer Genome Atlas and Gene Expression Omnibus and clustered the data into training (*n* = 490) and Validation (*n* = 226) datasets, respectively. To establish an epigenetic model, we identified prognostic epigenetic regulation-related genes by LASSO and Cox regression analyses, and established a novel 11-gene signature, including *EPC1, GADD45A, HCFC2, RCOR1, SMARCAL1, TLE2, TRIM28,* and *ZNF516*, for predicting LUAD overall survival (OS). The biosignature performed optimally in both the training and validation sets according to receiver operating characteristic and calibration plots. Moreover, the biosignature classified patients into high- and low-risk clusters with distinct survival times, with Cox regression analysis revealing the biosignature as an independent LUAD prognostic index. Furthermore, the generated nomogram integrating the prognostic gene biosignature and clinical indices predicted LUAD OS with high efficiency and outperformed tumor-node-metastasis staging in LUAD survival prediction. These results demonstrated the efficacy of the epigenetic signature prognostic nomogram for reliably predicting LUAD OS and its potential application for informing clinical decision making and individualized treatment.

## INTRODUCTION

Lung cancer is the leading cause of cancer-related mortality worldwide, with >1 million deaths reported annually [1]. Lung adenocarcinoma (LUAD), a major subclass of lung cancer, accounts for nearly 40% of lung cancer cases [2]. Despite considerable improvements in LUAD diagnosis and treatment, the prognosis for LUAD patients remains poor, with a 5-year survival rate ranging from ~10% to ~15%. Delayed diagnosis, disease relapse, and drug resistance are common causes of mortality in LUAD patients [3]. Although several prognostic models have provided insights for therapeutic strategies in lung cancer [4–6], predictive and prognostic signatures are needed to accurately diagnose and treat LUAD as a heterogeneous and complex disease.

Tumorigenesis is a multistep process involving genetic and epigenetic alterations [7]. Epigenetics is a fundamental regulatory mechanism of gene expression that involves DNA methylation, histone modification, noncoding RNA regulation, and chromatin remodeling [8–11]. Epigenetic abnormalities are reportedly involved in tumor initiation, progression, and recurrence [12, 13]. For example, aberrant methylation of DNA associated with genes encoding pathway molecules, such as those related to the extracellular-signal-regulated kinase (ERK) family, the Hedgehog signaling pathway, and the nuclear factor kappaB signaling pathway, were identified in lung squamous cell carcinoma by genome-wide association studies [14]. Additionally, epigenetic interplay between cancer, stromal, and immune cells in the tumor microenvironment play a vital role in both tumor initiation and progression. Inhibitors of histone deacetylases block monocyte-to-dendritic cell differentiation and result in a decreased immunogenic phenotype [15], with immune-cell evasion recognized as an emerging hallmark of cancer. These findings promote a deeper understanding of LUAD tumorigenesis and promote the development of potential epigenetic therapy.

However, to the best of our knowledge, the prognostic value of epigenetic regulation-related genes (ERGs) and their biological function in LUAD remain poorly defined. Here, we developed and validated a nomogram with an epigenetic signature for predicting prognosis in LUAD patients. We first identified ERGs related to LUAD prognosis and explored their potential functional mechanisms, followed by the development and validation of a nomogram with an epigenetic signature capable of predicting survival in LUAD patients. This study offers insight into the application of epigenetic signatures to improve the prognosis and clinical treatment of LUAD patients.

## RESULTS

### Construction of a prognostic model with a LUAD-specific epigenetic signature

**Figure 1:**
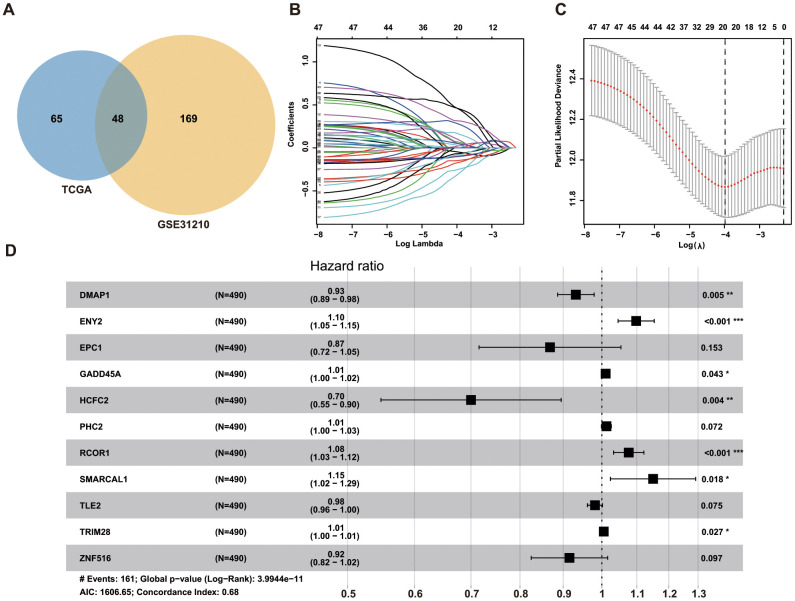
**Identification of ERGs for predicting survival of LUAD patients.** (**A**) Venn diagrams of prognostic ERGs in TCGA and GEO datasets. (**B**) Identification of 20 prognostic ERGs by LASSO regression analysis of TCGA data. (**C**) Each curve represents an ERG according to 1,000-fold cross-validation using 1-SE criteria in LASSO regression analysis. (**D**) Forrest plot of 11 ERGs generated by multivariate Cox regression analysis.

We first performed univariate Cox regression analysis to identify 113 and 217 prognosis-related ERGs in The Cancer Genome Atlas (TCGA) and GSE31210 datasets, respectively. Among these ERGs, we selected 48 that overlapped for further analysis (Figure 1A), and only 20 ERGs remained following LASSO Cox regression analysis of the training set (Figure 1B, 1C). We then performed stepwise forward multivariate Cox regression analysis to screen ERGs related to overall survival (OS), identifying 11 genes that were subsequently used to construct the prognostic model for LUAD patients (Figure 1D). A risk score for each patient was then calculated as follows: risk score = (−0.070819821 × *DMAP1* level) + (0.093606965 × *ENY2* level) + (−0.141271509 × *EPC1* level) + (0.01034072 × *GADD45A* level) + (−0.356532015 × *HCFC2* level) + (0.012487505 × *PHC2* level) + (0.073312056 × *RCOR1* level) + (0.139640667 × *SMARCAL1* level) + (−0.018668209 ×*TLE2* level) + (0.005275523 × *TRIM28* level) + (−0.088282786 × *ZNF516* level).

### ERG expression and genetic alteration in LUAD

**Figure 2:**
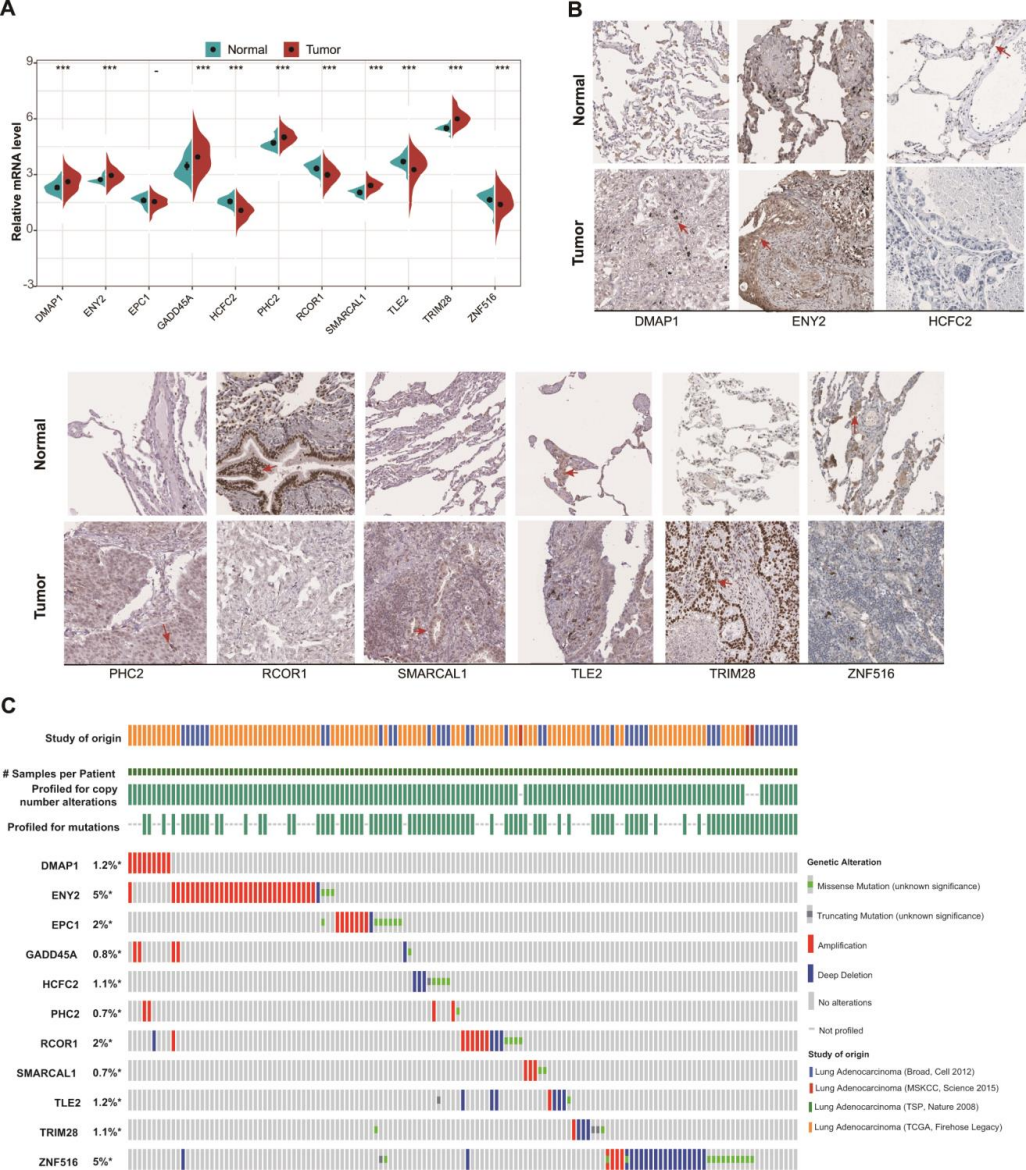
**mRNA and protein levels of and genetic alterations in the 11 identified ERGs.** (**A**) mRNA levels between tumor and normal tissues in the training set. (**B**) Protein levels between tumor and normal tissues obtained from the Human Protein Atlas database (HCFC2 and GADD45A are not available). (**C**) Genetic alterations in the 11 LUAD-related ERGs according to data obtained from the cBioPortal for Cancer Genomics.

We then evaluated mRNA levels of the 11 ERGs between tumor tissues and normal lung tissue. We found that *DMAP1*, *ENY2*, *GADD45A*, *PHC2*, *SMARCAL1*, and *TRIM28* expression was significantly elevated and *HCFC2*, *RCOR1*, and *TLE2* expression significantly decreased in tumor tissue relative to normal tissue, with no difference in EPC1 expression observed between tissue types (Figure 2A). Analysis of protein levels for the 11 ERGs agreed with mRNA results (Figure 2B). Additionally, we evaluated genetic alterations in the 11 ERGs across four LUAD datasets, with the most commonly identified changes being mutations, amplifications, and deletions found in only 0.7% to 5% of the genes (Figure 2C).

### Gene set enrichment analysis (GSEA) and gene set variation analysis (GSVA)

**Figure 3:**
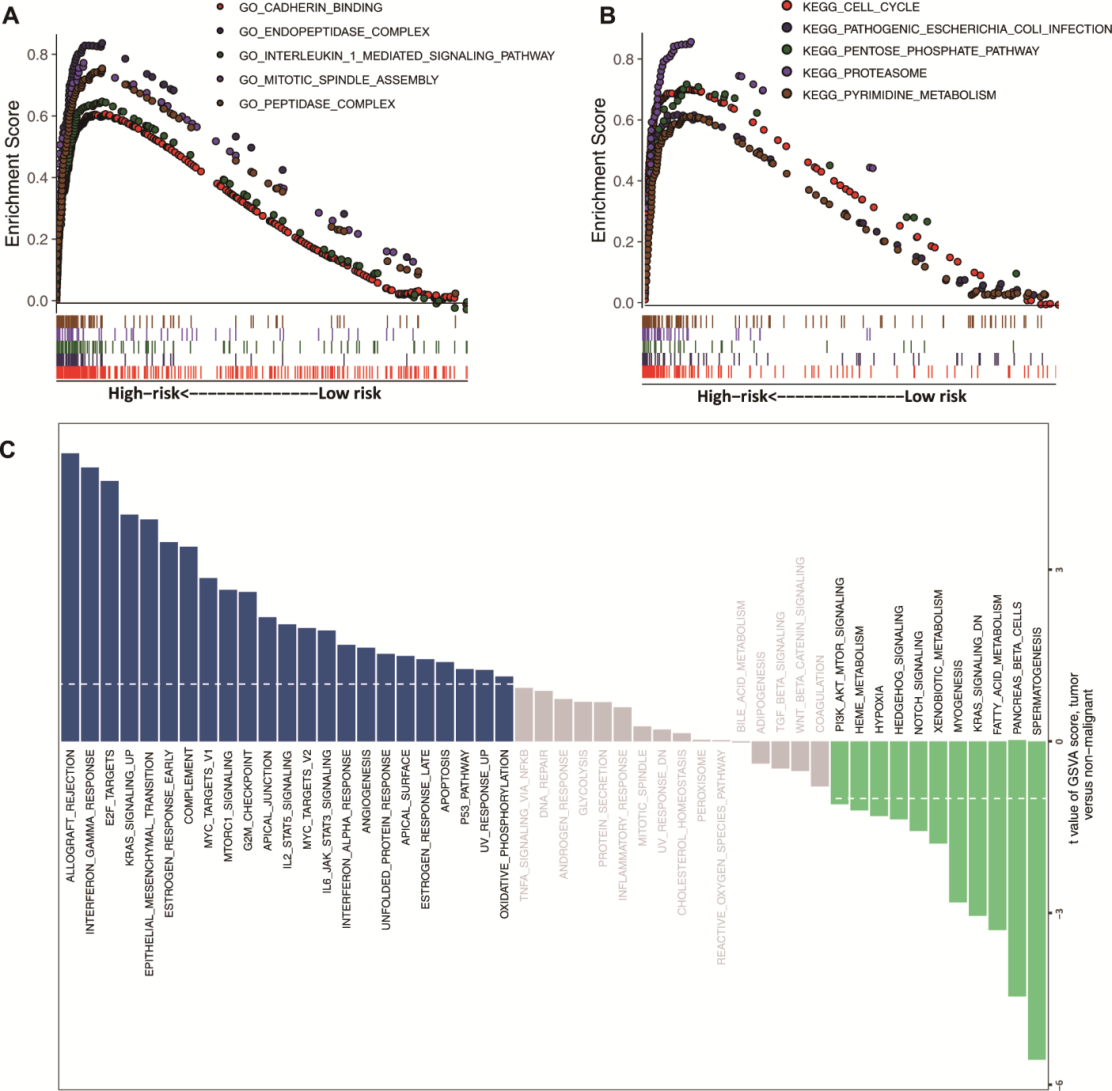
**GSEA and GSVA.** Top 5 representative (**A**) GO terms and (**B**) enriched KEGG pathways between high- and low-risk groups. (**C**) GSVA of the high- and low-risk clusters.

We then performed functional enrichment analysis between high- and low-risk groups. The results indicated that the top 5 Gene Ontology (GO) terms and Kyoto Encyclopedia of Genes and Genomes (KEGG) pathways were significantly enriched for the high-risk phenotype (cadherin binding, peptidase complex, interleukin 1-mediated signaling pathway, cell cycle, proteasome, and pyrimidine metabolism) (Figure 3A, 3B). Additionally, GSVA revealed that the epithelial-to-mesenchymal transition, the G2M checkpoint, angiogenesis, and the p53 pathway were significantly activated in the high-risk group (Figure 3C). These results suggested that tumorigenesis-related pathways were enriched in the high-risk group.

### Prognostic significance of the epigenetic biosignature in the training set

**Figure 4:**
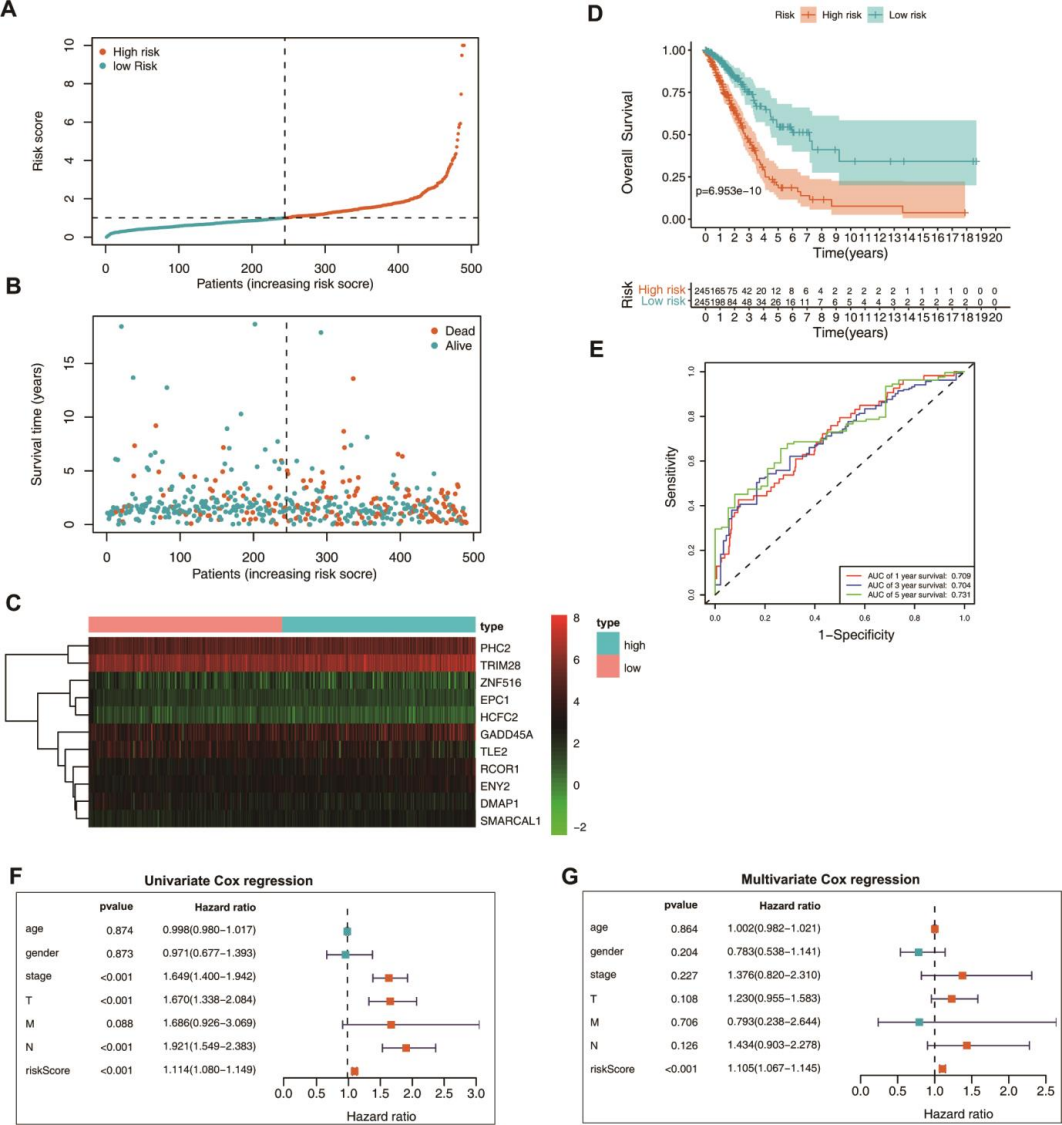
**Prognostic value of the epigenetic signature using the training set.** (**A**) Rankings for the risk signature and group distribution. (**B**) Survival status of patients in the low- and high-risk groups. (**C**) Heatmap of the gene-expression profiles. (**D**) Patients in the high-risk group demonstrated poor OS. (**E**) ROC curve showing the prognostic significance of the risk signature. (**F**) Univariate and (**G**) multivariate Cox regression analyses of discrete clinical factors.

Patient data included in the training set were clustered into high- (*n* = 245) and low-risk clusters (*n* = 245) according to the median risk score, with the risk-score distribution shown in Figure 4A. Patients in the high-risk group displayed a worse OS relative to those in the low-risk group (Figure 4B, 4D). Additionally, area under the receiver operating characteristic (ROC) curve (AUC) values generated to predict 1-, 3-, and 5-year survival were 0.709, 0.704, and 0.731, respectively (Figure 4E), indicating that this epigenetic biosignature showed good predictive capability. Moreover, Cox regression analysis demonstrated the biosignature as an independent predictor following adjustment of clinicopathological features, including age, sex, grade, and tumor-node-metastasis (TNM) stage (Figure 4F, 4G).

### Verification of the epigenetic biosignature in the validation set

**Figure 5:**
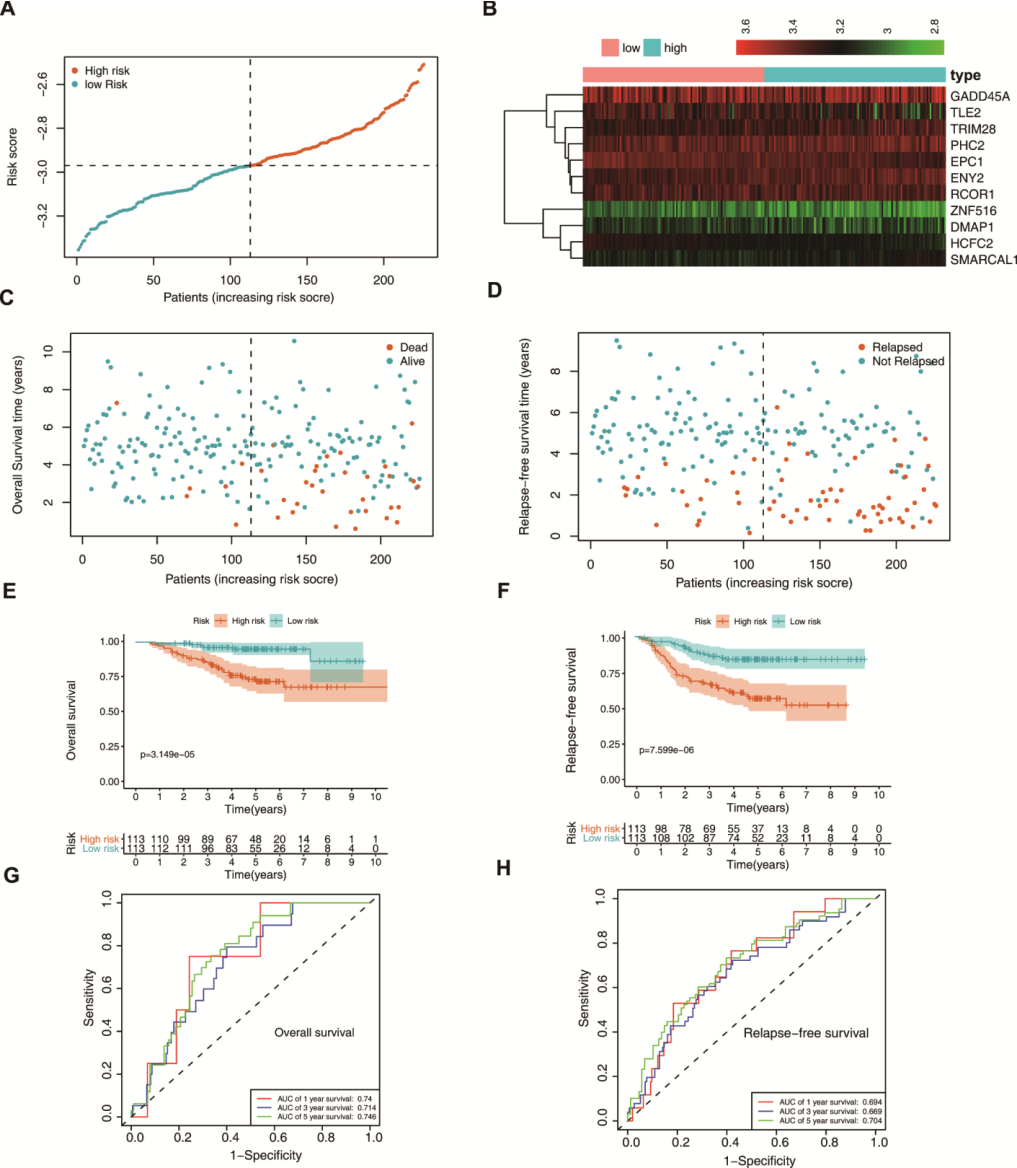
**Validation of the epigenetic biosignature in the test set.** (**A**) Rankings for the risk signature and group distribution. (**B**) Heatmap of the gene-expression profiles. Patients in the high-risk group demonstrated (**C**) earlier mortality and (**D**) earlier relapse. (**E**) OS and (**F**) RFS of patients in the low- and high-risk groups. ROC analyses of (**G**) OS and (**H**) RFS predictions using the epigenetic signature.

We then verified the predictive potential of the epigenetic biosignature using the GSE39582 dataset. Figure 5A through 5D shows the risk-score distribution, survival status, and a heatmap of the 10 ERG expression profiles between the high- and low-risk groups. Survival analysis revealed that OS and relapse-free survival (RFS) were markedly lower in the high-risk group (Figure 5E, 5F), which was consistent with findings using the training set and demonstrated that the epigenetic biosignature could discriminate the high-risk group from overall LUAD patients. Additionally, the AUC values showed good accuracy in prognostic predictions of patient survival (Figure 5G, 5H), confirming the good predictive performance of the signature for LUAD patient survival.

### Correlation between the signature and clinicopathological features

**Figure 6:**
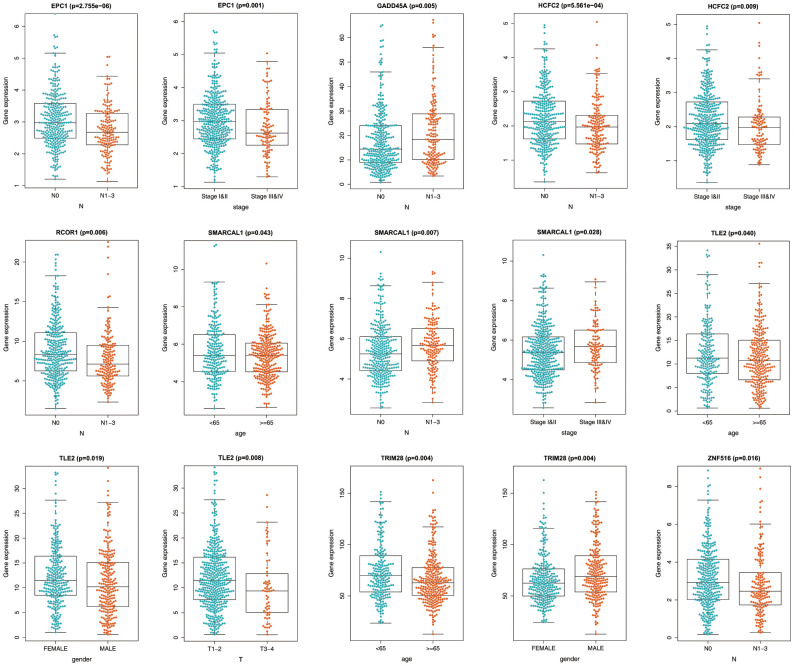
**Relationship between the prognostic epigenetic biosignature and clinicopathological features.**

We then analyzed correlations between the epigenetic signature and clinicopathological features, including age, gender, pathological stage, and TNM stage, in the training set. We found that *TRIM28* mRNA level was significantly elevated in males, whereas *TLE2* level was significantly lower. Additionally, mRNA levels of *SMARCAL1*, *TLE2*, and *TRIM28* were lower among patients aged ≥65 years, and differential expression of *EPC1*, *GADD45A*, *HCFC2*, *RCOR1*, *SMARCAL1*, *TLE2*, and *ZNF516* was observed in patients exhibiting different pathological and TNM stages (Figure 6). These results suggested that the epigenetic biosignature was closely related to various clinicopathological features.

### Subgroup analysis of the prognostic significance of the epigenetic signature

**Figure 7:**
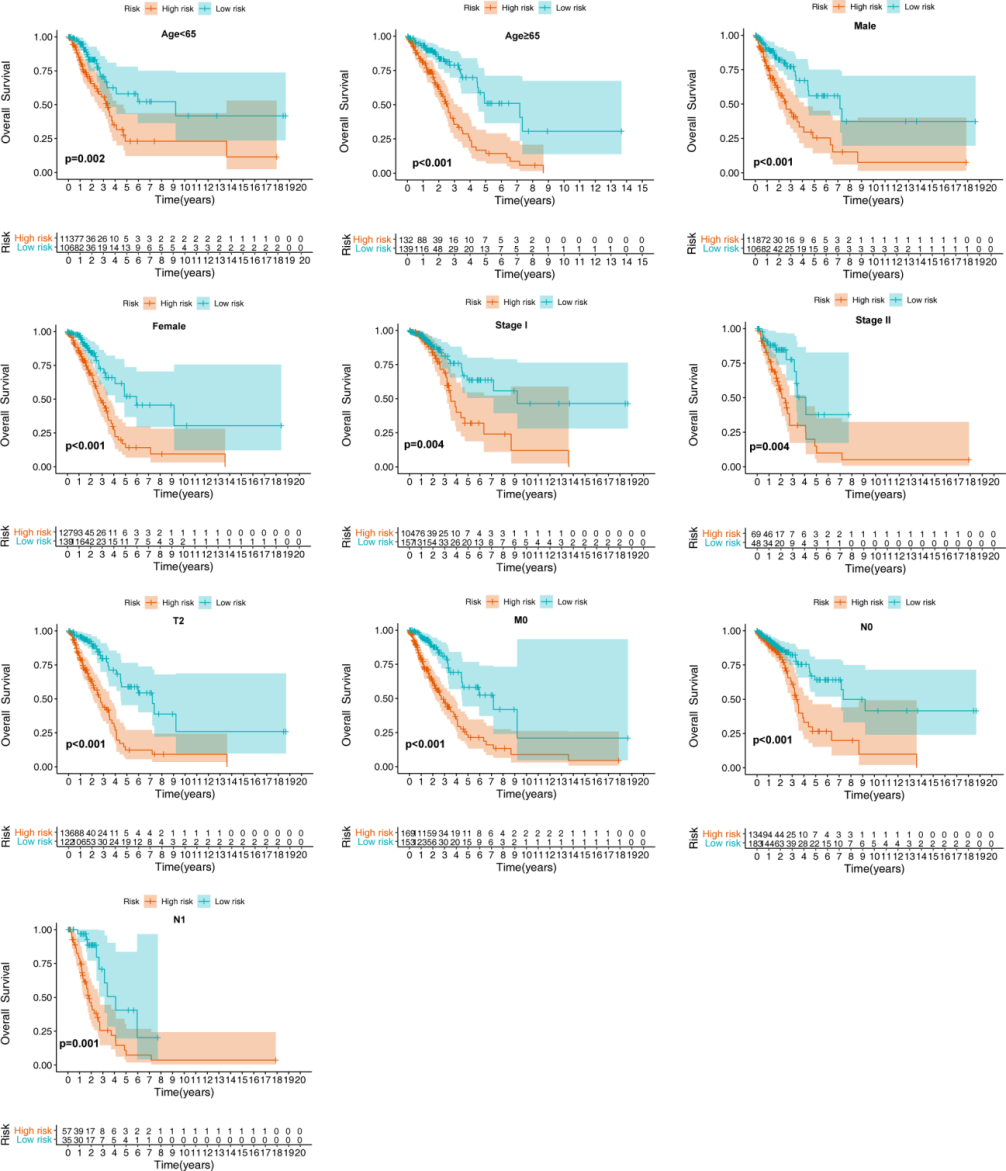
**Verification of the biosignature stratified by different clinical parameters in the training set.**

Given the link between the ERG-related biosignature and clinicopathological features, we evaluated whether the prognostic significance of the model was suitable for other clinical parameters. Using the training set, the model accurately predicted OS between low- and high-risk groups in subclusters including patients exhibiting various clinicopathological features, including age, gender, cancer stages (I and II, T2, N0-1, and M0) (Figure 7 and Table 1). Additionally, the model accurately predicted OS and RFS between the low- and high-risk groups in subclusters including patients of various ages and genders, as well as smoking status, cancer stage, presence of epidermal growth factor receptor (*EGFR*) mutation, and those with EGFR/KRAS/anaplastic lymphoma kinase (ALK)-negative LUAD (Table 2).

**Table 1:** The association between the signature and OS of LUAD patients in training set (n=490).

**Characteristics**	**Number (low/high)**	**Percentage (%)**	**HR (95%CI) (low/high)**	**P-value**
**Age(years)**				
≥65	132/139	55.3%	3.127(2.014-4.857)	0.000
<65	113/106	44.7%	2.313(1.375-3.890)	0.002
**Gender**				
Female	127/139	54.3%	2.700(1.687-4.320)	0.000
Male	118/106	45.7%	2.633(1.626-4.264)	0.000
**Stage**				
I	104/157	53.3%	2.238(1.300-3.852)	0.004
II	69/48	23.9%	2.682(1.362-5.284)	0.004
III	54/25	16.1%	1.936(0.921-4.070)	0.082
IV	15/10	5.1%	1.718(0.532-5.550)	0.366
NA	3/5	1.6%	-	-
**T stage**				
T1	69/97	33.9%	1.662(0.885-3.120)	0.114
T2	136/122	52.7%	3.634(2.277-5.799)	0.000
T3	29/16	9.2%	2.223(0.712-6.940)	0.169
T4	10/8	3.7%	2.720(0.599-13.237)	0.215
NA	1/2	0.6%	-	-
**M stage**				
M0	169/153	65.7%	3.192(2.053-4.961)	0.000
M1	15/9	4.9%	2.110(0.574-7.754)	0.261
NA	61/83	29.4%	-	-
**N stage**				
N0	134/183	64.7%	2.368(1.481-3.788)	0.000
N1	57/35	18.8%	3.481(1.680-7.210)	0.001
N2	49/19	13.9%	1.598(0.724-3.531)	0.246
N3	2/0	0.4%	-	-
NA	3/8	2.2%	-	-

NA, not available.

**Table 2:** The association between epigenetic signature and survival (OS and RFS) of LUAD patients in validation set (n=266).

**Characteristics**	**Number (low/high)**	**Percentage (%)**	**OS (low/high)**		**RFS (low/high)**	
			**HR (95%CI)**	**P-value**	**HR (95%CI)**	**P-value**
**Age(years)**						
≥65	33/29	27.4%	4.736(1.314-17.07)	0.017	3.879(1.516-9.925)	0.005
<65	80/84	72.6%	5.303(2.201-12.78)	0.000	3.308(1.898-5.765)	0.000
**Gender**						
Female	66/55	53.5%	2.680(0.930-7.723)	0.068	1.957(0.966-3.964)	0.062
Male	47/58	46.5%	16.64(2.22-124.718)	0.006	7.086(2.483-20.225)	0.000
**Smoke status**						
Ever smoker	47/64	49.1%	15.177(2.031-113.402)	0.008	5.193(2.008-13.427)	0.001
Never smoker	66/49	50.9%	2.037(0.980-4.236)	0.057	2.285(1.100-4.748)	0.027
**Stage**						
I	99/69	74.3%	7.295(2.094-25.420)	0.002	3.465(1.738-6.905)	0.000
II	14/44	25.7%	1.503(0.434-5.202)	0.520	1.280(0.483-3.391)	0.619
**Mutation**						
ALK-fusion+	5/6	4.9%	0.745(0.046-11.968)	0.836	0.645(0.039-10.556)	0.645
EGFR mutation+	68/59	56.2%	15.974(2.098-122.148)	0.008	3.740(1.664-8.408)	0.001
KRAS mutation+	7/13	8.8%	38.256(0.001-113.116)	0.488	3.028(0.353-25.951)	0.312
EGFR/KRAS/ALK-	33/35	30.1%	3.758(1.206-11.695)	0.022	3.587(1.499-8.584)	0.004

### Immune-cell profiles in low- and high-risk groups

**Figure 8:**
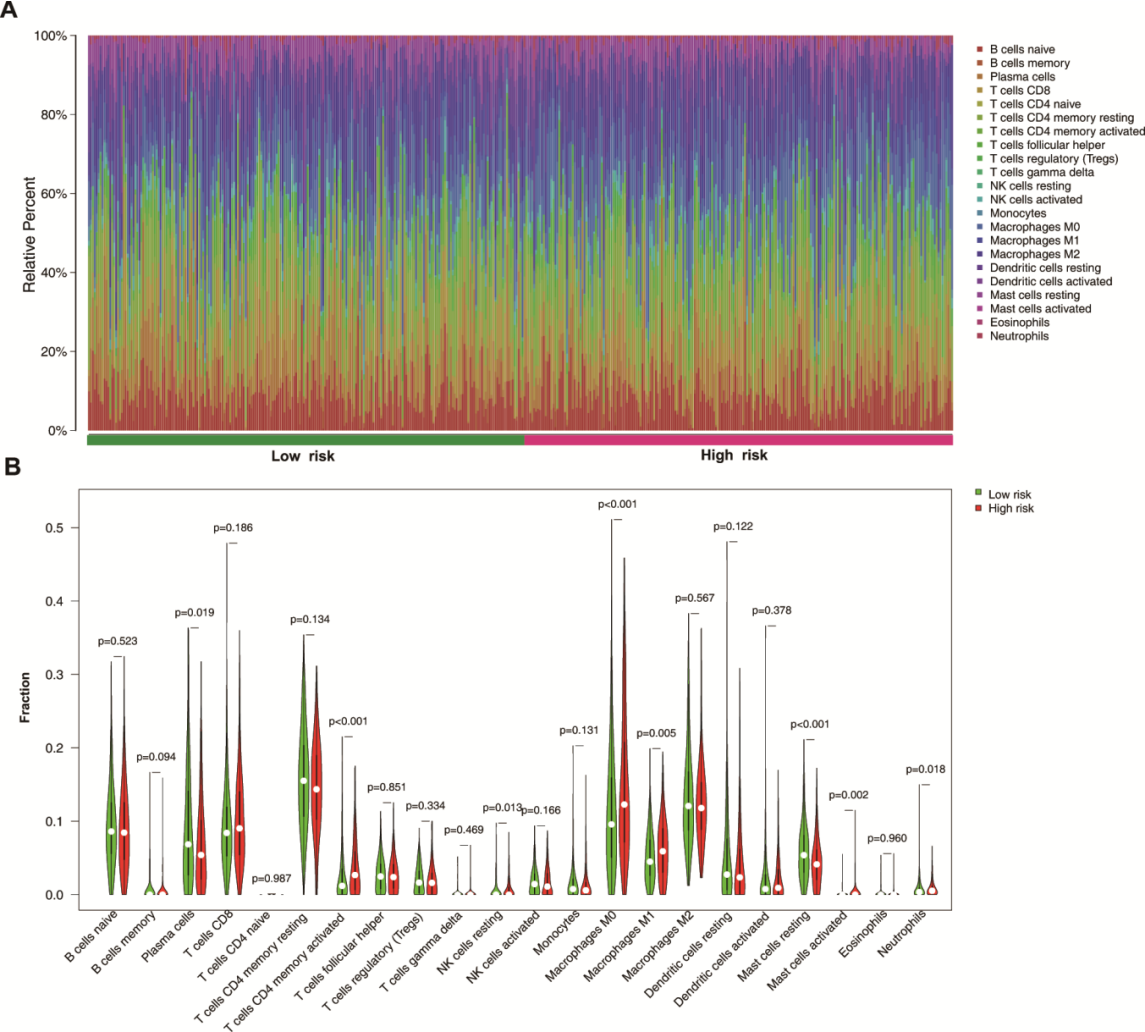
**Immune-cell distribution between low- and high-risk groups.** (**A**) Relative proportion of immune cells between two groups. (**B**) Violin plots immune-cell distribution between groups.

We then investigated the abundance of infiltrated immune cells in tumor tissues between high- and low- risk groups. The results revealed that the high-risk group showed higher proportions of activated memory CD4^+^ T cells, resting natural killer (NK) cells, M0 and M1 macrophages, activated mast cells, and neutrophils but lower levels of plasma cells and resting mast cells (Figure 8A, 8B).

### Nomogram construction and validation of nomogram

**Figure 9:**
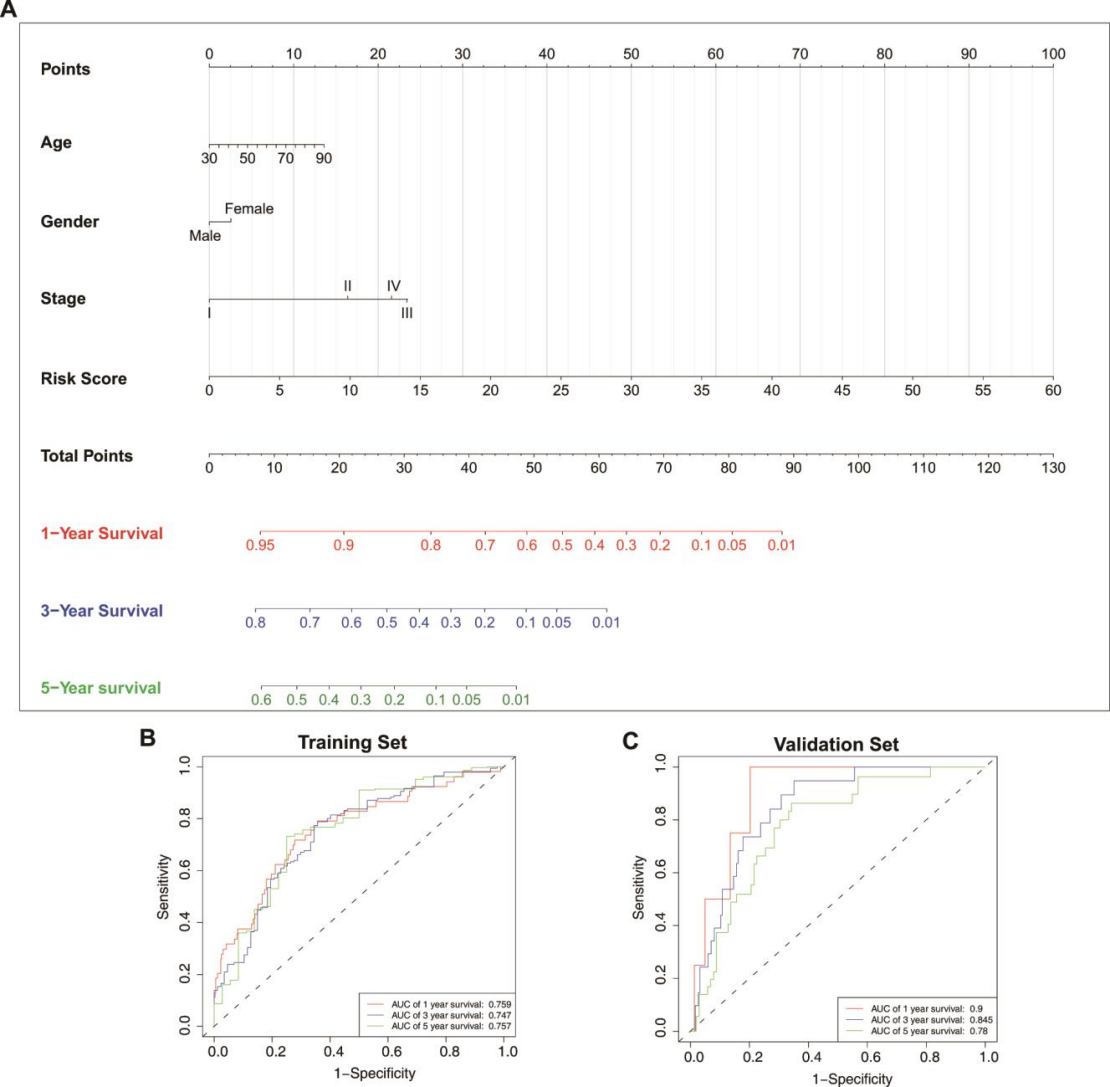
**Nomogram construction and validation.** (**A**) Nomogram generated based on the epigenetic signature and clinical traits. ROC curves for nomogram-based prognostic prediction using the (**B**) training and (**C**) test sets.

**Figure 10:**
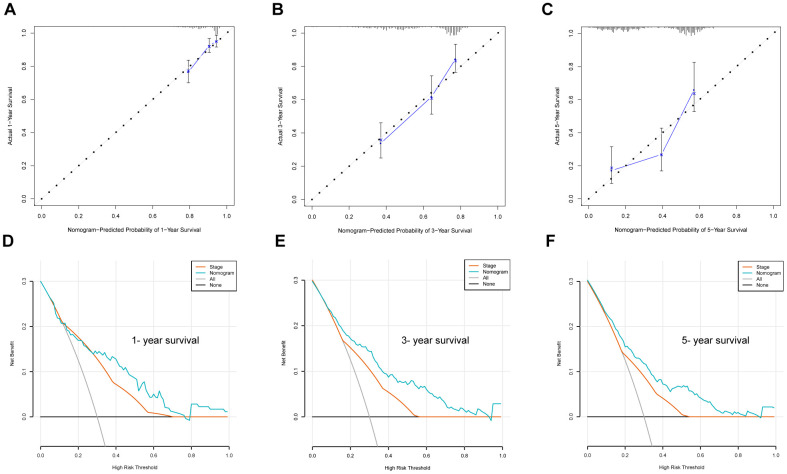
**Nomogram evaluation using the training set.** (**A–C**) Calibration plot examining the estimation accuracy. (**D–F**) DCA assessing clinical utility.

To predict the OS of LUAD patients, we generated a nomogram incorporating the ERG biosignature, pathological stage, age, and gender using the training set (Figure 9A). AUC values for predicting 1-, 3-, and 5-year survival were 0.759, 0.747, and 0.757, respectively (Figure 9B), and those for 1-, 3-, and 5-year survival probability were 0.9, 0.845, and 0.78, respectively (Figure 9C). Additionally, ROC results indicated that the nomogram showed good predictive value, and calibration plots confirmed accurate estimation of 1-, 3-, and 5-year OS using the training set (Figure 10A–10C). Furthermore, decision curve analysis (DCA) suggested the clinical utility of the nomogram for predicting LUAD patient prognosis (Figure 10D–10F). These results demonstrated that the nomogram outperformed the use of single independent risk factors in predictive performance.

## DISCUSSION

Most of the established biomarkers used for LUAD treatment response and survival are based on clinical indices with limited accuracy and specificity. Genomic and transcriptomic analyses have provided a comprehensive understanding of genetic and epigenetic alterations in cancer. Previous studies have reported the utility of epigenetic signatures as prognostic indicators in breast and colon cancers [16, 17]; however, the efficacy of such a signature as an independent prognostic factor for LUAD has not been determined. In the present study, we developed an epigenetic signature based on 11 ERGs (*DMAP1, ENY2, EPC1, GADD45A,* *HCFC2, PHC2, RCOR1, SMARCAL1, TLE2, TRIM28,* and *ZNF516*) and constructed a nomogram for predicting LUAD patient survival. The results suggested that this epigenetic signature could differentiate between low- and high-risk groups, and that the nomogram could serve as a reliable tool for predicting LUAD patient survival.

The majority of ERGs included in our signature are closely related to tumor initiation, proliferation, and metastasis. Yamaguchi et al. [18] reported that low expression of *DMAP1* is related to poor prognosis in neuroblastoma patients and contributes to tumorigenesis through inhibition of ataxia telangiectasia mutated/p53 pathway activation. ENY2, a nuclear transcription factor, coordinates the activity of multiple H2B deubiquitinases, thereby potentiating tumor proliferation and growth [19]. Additionally, Wang et al. [20] identified a novel oncogenic function of EPC1 that involves activation of metastasis-related gene expression. A previous study described GADD45A as a tumor suppressor capable of inducing G2/M phase arrest and apoptosis [21]. Wang et al. [22] reported that hypermethylation of PHC2 is associated with prostate carcinogenesis, and Xiang et al. [23] showed that RCOR1 directly binds to MED28 to weaken its induction of cancer stem cell-like activity in carcinoma cells. SMARCAL, a chromatin remodeling factor, decreases telomere-replication stress related to carcinogenesis [24, 25], and TLE2 is highly expressed in patients with early stage bladder cancer and correlates with favorable prognosis [26]. Furthermore, TRIM28, a transcriptional corepressor, reportedly promotes tumor proliferation and metastasis [27, 28]. There are limited studies of the tumor specific roles of HCFC2 and ZNF516, suggesting that additional studies are needed to elucidate their associations with LUAD.

Using these 11 ERGs, we applied an epigenetic signature as an independent prognostic factor for LUAD patients using several survival-analysis methods and successfully distinguished low- and high-risk groups. Additionally, we found that this signature was suitable for risk assessment in LUAD patients with different clinicopathological traits, including age, sex, pathological stage, TNM stage, and gene-mutation status. These clinical features were previously confirmed as closely associated with LUAD patient prognosis [29–31]. The generated nomogram incorporated both the epigenetic signature and clinical indices to predict LUAD patient survival, resulting in a predictive accuracy confirmed using ROC and calibration plots. The findings suggested its reliability as a tool for individualized assessment of LUAD survival and a promising strategy for LUAD management.

Additionally, we explored the differential distribution of infiltrating immune cells in the tumor microenvironment between low- and high-risk groups. The results revealed that proportions of activated memory CD4+ T cells, resting NK cells, M0 and M1 macrophages, activated mast cells, and neutrophils were higher in the high-risk group relative to those in the low-risk group, indicating a correlation between signature-specific prediction of LUAD survival and immune-cell infiltration. Epigenetic alterations such as DNA methylation play a ubiquitous role in regulation of immune cells function. Evidence revealed that epigenetic programming is associated with macrophage polarization and T cell differentiation [32, 33]. M0 and M1 macrophages secrete proinflammatory cytokines that trigger chronic inflammation locally and systemically and epigenetic therapy also could induce the secretion of these cytokines, thereby promoting tumor progression or initiating cancer immunotherapy [34]. In addition, Li et al. [35] reported that histone demethylase Jmjd3 ablation promotes CD4+ T cell differentiation into Th2 and Th17 cells. These results provide insight into immunological and epigenetic processes associated with LUAD.

One study limitation is that other risk factors for LUAD, such as emphysema and chronic obstructive pulmonary disease, were not collected from TCGA or Gene Expression Omnibus (GEO) datasets. Further research should be undertaken to validate this model in larger LUAD cohorts. Furthermore, *in vitro* or *in vivo* experiments are needed to investigate the underlying mechanisms associated with the prognostic significance of the identified ERGs in LUAD.

In summary, we constructed and validated a nomogram incorporating an epigenetic signature and clinical traits of patients (age, gender, and TNM stage) for predicting the survival in LUAD patients. This nomogram could serve as a reliable tool for determining LUAD treatment strategies and potential outcomes.

## MATERIALS AND METHODS

### Data collection

Gene-expression profiles from LUAD tissues were downloaded from TCGA (https://portal.gdc.cancer.gov) and GEO (GSE31210 [36]; https://www.ncbi.nlm.nih.gov/geo/) and used as training and testing datasets, respectively. The GSE31210 dataset includes 226 frozen tissue of primary lung tumors from patients with lung adenocarcinomas based on the GPL570 (Affymetrix Human Genome U133A 2.0 Array) platform. Samples with incomplete survival data or follow-up times of <1 day were excluded, resulting in 490 LUAD cases from TCGA database used for analysis. An ERG list was obtained from EpiFactors (http://epifactors.autosome.ru/) [37], and protein expression of the ERGs in LUAD and non-cancerous tissues was assessed using the Human Protein Atlas (https://www.proteinatlas.org/). ERG mutation data were acquired from the cBioPortal for Cancer Genomics (https://www.cbioportal.org/).

### Development and validation of an ERG prognostic signature

We first screened prognosis-related genes in the overall cohort (*n* = 716) using univariate Cox and LASSO regression analyses. Multivariate Cox regression analysis was subsequently used to identify independent prognostic parameters in the training set (*n* = 490). Risk scores were calculated for each patient in both the training and test sets based on gene-expression levels and coefficients of multivariate Cox regression. The patients were then clustered into high- and low-risk group based on their median risk score. Kaplan–Meier analysis was performed to generate curves using the log rank test in order to assess differences in survival between the high- and low-risk groups. Additionally, ERG expression levels were analyzed between groups, and Kaplan–Meier analysis was performed to evaluate survival according to various clinicopathological characteristics.

### GSEA and GSVA

GSEA (http://software.broadinstitute.org/gsea/index.jsp) was used to explore potential biological functions and enriched pathways between high- and low-risk groups in the training set. The normalized enrichment score was obtained from 1,000 permutations. Additionally, GSVA was performed to evaluate differential pathway activation between high- and low-risk groups using the “GSVA” R package (https://www.r-project.org/). A cut-off criterion of *P* < 0.05 was considered statistically significant.

### Immune-cell analysis

We assessed 22 immune-cell types, including both innate and adaptive immune cells, in the low- and high-risk groups using the CIBERSORT algorithm (https://cibersort.stanford.edu/). To improve the reliability of the deconvolution method, samples with a CIBERSORT *P* < 0.05 were selected for further analysis. The number of permutations was set at 100.

### Nomogram development and validation

We constructed a nomogram using patient risk scores and clinical indices (age, gender, and TNM/pathological stage), and calibration plots were generated to test the performance of the predictive nomogram using the training set. Additionally, we performed ROC analysis to examine the predictive accuracy of the nomogram by internal (training set) and external (verification set) validation. DCA was performed to evaluate the clinical usefulness of the nomogram.

### Statistical analysis

mRNA-expression profiles from TCGA and GEO datasets were extracted as raw data, with expression levels normalized by log2 transformation. All statistical analyses were conducted in R (v.3.6.2; https://www.r-project.org/), and a *P* < 0.05 was considered statistically significant.
